# Quantification of myocardial extracellular volume without blood sampling

**DOI:** 10.1093/ehjimp/qyad022

**Published:** 2023-09-15

**Authors:** Wensu Chen, Alessandro Faragli, Collin Goetze, Victoria Zieschang, Karl Jakob Weiss, Djawid Hashemi, Rebecca Beyer, Lorena Hafermann, Philipp Stawowy, Sebastian Kelle, Patrick Doeblin

**Affiliations:** Department of Cardiology, Angiology and Intensive Care Medicine, Deutsches Herzzentrum der Charité, Augustenburger Platz 1, Berlin 13353, Germany; Charité – Universitätsmedizin Berlin, corporate member of Freie Universität Berlin and Humboldt-Universität zu Berlin, Charitéplatz 1, Berlin 10117, Germany; Department of Cardiology, Affiliated Hospital of Xuzhou Medical University, Xuzhou, China; Department of Cardiology, Angiology and Intensive Care Medicine, Deutsches Herzzentrum der Charité, Augustenburger Platz 1, Berlin 13353, Germany; Charité – Universitätsmedizin Berlin, corporate member of Freie Universität Berlin and Humboldt-Universität zu Berlin, Charitéplatz 1, Berlin 10117, Germany; Berlin Institute of Health at Charité—Universitätsmedizin Berlin, Charitéplatz 1, Berlin 10117, Germany; DZHK (German Center for Cardiovascular Research), Partner Site Berlin, Potsdamer Str. 58, Berlin 10785, Germany; Department of Cardiology, Angiology and Intensive Care Medicine, Deutsches Herzzentrum der Charité, Augustenburger Platz 1, Berlin 13353, Germany; Charité – Universitätsmedizin Berlin, corporate member of Freie Universität Berlin and Humboldt-Universität zu Berlin, Charitéplatz 1, Berlin 10117, Germany; Department of Cardiology, Angiology and Intensive Care Medicine, Deutsches Herzzentrum der Charité, Augustenburger Platz 1, Berlin 13353, Germany; Charité – Universitätsmedizin Berlin, corporate member of Freie Universität Berlin and Humboldt-Universität zu Berlin, Charitéplatz 1, Berlin 10117, Germany; Department of Cardiology, Angiology and Intensive Care Medicine, Deutsches Herzzentrum der Charité, Augustenburger Platz 1, Berlin 13353, Germany; Charité – Universitätsmedizin Berlin, corporate member of Freie Universität Berlin and Humboldt-Universität zu Berlin, Charitéplatz 1, Berlin 10117, Germany; DZHK (German Center for Cardiovascular Research), Partner Site Berlin, Potsdamer Str. 58, Berlin 10785, Germany; Department of Cardiology, Angiology and Intensive Care Medicine, Deutsches Herzzentrum der Charité, Augustenburger Platz 1, Berlin 13353, Germany; Charité – Universitätsmedizin Berlin, corporate member of Freie Universität Berlin and Humboldt-Universität zu Berlin, Charitéplatz 1, Berlin 10117, Germany; DZHK (German Center for Cardiovascular Research), Partner Site Berlin, Potsdamer Str. 58, Berlin 10785, Germany; Department of Cardiology, Angiology and Intensive Care Medicine, Deutsches Herzzentrum der Charité, Augustenburger Platz 1, Berlin 13353, Germany; Charité – Universitätsmedizin Berlin, corporate member of Freie Universität Berlin and Humboldt-Universität zu Berlin, Charitéplatz 1, Berlin 10117, Germany; Institute of Biometry and Clinical Epidemiology, Charité – Universitätsmedizin Berlin, corporate member of Freie Universität Berlin and Humboldt-Universität zu Berlin, Charitéplatz 1, Berlin 10117, Germany; Department of Cardiology, Angiology and Intensive Care Medicine, Deutsches Herzzentrum der Charité, Augustenburger Platz 1, Berlin 13353, Germany; Charité – Universitätsmedizin Berlin, corporate member of Freie Universität Berlin and Humboldt-Universität zu Berlin, Charitéplatz 1, Berlin 10117, Germany; DZHK (German Center for Cardiovascular Research), Partner Site Berlin, Potsdamer Str. 58, Berlin 10785, Germany; Department of Cardiology, Angiology and Intensive Care Medicine, Deutsches Herzzentrum der Charité, Augustenburger Platz 1, Berlin 13353, Germany; Charité – Universitätsmedizin Berlin, corporate member of Freie Universität Berlin and Humboldt-Universität zu Berlin, Charitéplatz 1, Berlin 10117, Germany; Berlin Institute of Health at Charité—Universitätsmedizin Berlin, Charitéplatz 1, Berlin 10117, Germany; DZHK (German Center for Cardiovascular Research), Partner Site Berlin, Potsdamer Str. 58, Berlin 10785, Germany; Department of Cardiology, Angiology and Intensive Care Medicine, Deutsches Herzzentrum der Charité, Augustenburger Platz 1, Berlin 13353, Germany; Charité – Universitätsmedizin Berlin, corporate member of Freie Universität Berlin and Humboldt-Universität zu Berlin, Charitéplatz 1, Berlin 10117, Germany; DZHK (German Center for Cardiovascular Research), Partner Site Berlin, Potsdamer Str. 58, Berlin 10785, Germany

**Keywords:** CMR, *T*1 mapping, synthetic HCT, ECV classification description, cardiac magnetic resonance (CMR), tissue characterization

## Abstract

**Aims:**

Cardiac magnetic resonance (CMR) *T*1 relaxation time mapping is an established technique primarily used to identify diffuse interstitial fibrosis and oedema. The myocardial extracellular volume (ECV) can be calculated from pre- and post-contrast *T*1 relaxation times and is a reproducible parametric index of the proportion of volume occupied by non-cardiomyocyte components in myocardial tissue. The conventional calculation of the ECV requires blood sampling to measure the haematocrit (HCT). Given the high variability of the HCT, the blood collection is recommended within 24 h of the CMR scan, limiting its applicability and posing a barrier to the clinical routine use of ECV measurements. In recent years, several research groups have proposed a method to determine the ECV by CMR without blood sampling. This is based on the inverse relationship between the *T*1 relaxation rate (*R*1) of blood and the HCT. Consequently, a ‘synthetic’ HCT could be estimated from the native blood *R*1, avoiding blood sampling.

**Methods and results:**

We performed a review and meta-analysis of published studies on synthetic ECV, as well as a secondary analysis of previously published data to examine the effect of the chosen regression modell on bias. While, overall, a good correlation and little bias between synthetic and conventional ECV were found in these studies, questions regarding its accuracy remain.

**Conclusion:**

Synthetic HCT and ECV can provide a ‘non-invasive’ quantitative measurement of the myocardium’s extracellular space when timely HCT measurements are not available and large alterations in ECV are expected, such as in cardiac amyloidosis. Due to the dependency of *T*1 relaxation times on the local setup, calculation of local formulas using linear regression is recommended, which can be easily performed using available data.

## Graphical Abstract

**Graphical abstract:**
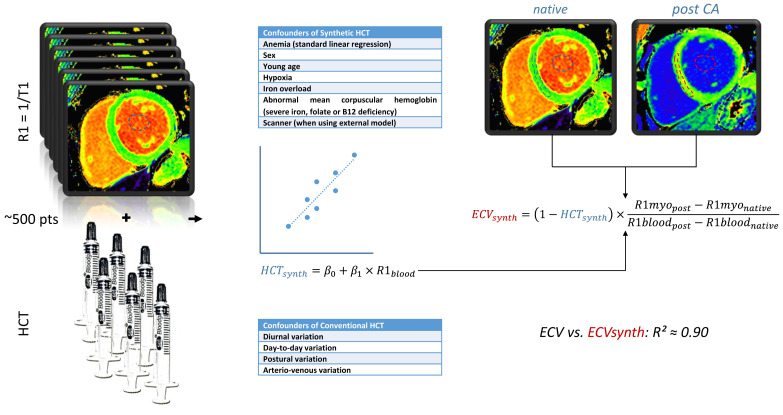
For the establishment of a local model, ∼500 patients with measured haematocrit (HCT) and left ventricular *T*1 relaxation rate (*R*1 = 1/*T*1) should be available and split evenly into derivation and validation data sets. The regression parameters from the derivation data is used for the estimation of synthetic HCT, which is then used for the calculation of the synthetic extracellular volume (ECV) instead of the measured HCT.

## Introduction

### Myocardial fibrosis

Myocardial fibrosis is a frequent finding in various cardiac pathologies and a marker of disease severity and adverse prognosis.^1^ While histopathological analysis using endomyocardial biopsy is considered the gold standard, cardiac magnetic resonance (CMR) imaging is increasingly used as a non-invasive alternative in research and clinical work for the assessment of myocardial fibrosis. The main CMR techniques to identify and quantify myocardial fibrosis by CMR are late gadolinium enhancement (LGE), *T*1 mapping, and the calculation of the extracellular volume (ECV). While LGE readily identifies focal fibrosis and scar, it may underestimate diffuse myocardial fibrosis as it requires healthy myocardium as a reference. Diseases that are histologically characterized by interstitial myocardial fibrosis, such as amyloidosis, hypertrophic cardiomyopathy, dilated cardiomyopathy, or myocarditis, may present with varying combinations of focal and diffuse fibrosis.^2–6^ Native *T*1 relaxation time mapping can identify diffuse interstitial fibrosis but is non-specific and does not quantify the extent of fibrosis.^7^ Pre- and post-contrast *T*1 mapping allows for the calculation of the ECV, a quantitative and specific measurement of diffuse myocardial fibrosis without the need for healthy ‘reference’ myocardium that is increasingly used to complement LGE imaging.

### *T*1 mapping

*T*1 mapping refers to the measurement of *T*1 relaxation time, or spin-lattice relaxation time, on a voxel-by-voxel basis over the entire field of view. The *T*1 relaxation time is the time at which a tissue recovers ∼63% of its longitudinal relaxation (parallel to the main magnetic field). Several *T*1 mapping techniques have been proposed, with the Modified Look–Locker Inversion (MOLLI) sequence having the widest adoption. The *T*1 relaxation time of tissues is dependent on the magnetic field strength of the scanner. A recent meta-analysis aggregating data from 120 publications and 5541 healthy subjects found a mean myocardial native *T*1 relaxation time of 976 ms [95% confidence interval (CI) for the mean: 969–983 ms] at 1.5 T and 1159 ms (95% CI: 1143–1175 ms) at 3 T for MOLLI-based pulse sequences.^8^ Notably, there was a high degree of heterogeneity in *T*1 time, with variations between vendors, MOLLI pulse sequence acquisition strategies, and sequence parameters including flip-angle and inter-inversion pulse delay. Furthermore, elevations in *T*1 time are not specific for fibrosis and are also encountered in oedema.

### ECV

The wide variability of *T*1 reference values between centres and scanners complicates its interpretation. ECV on the other hand is a relatively stable parameter based on pre- and post-contrast *T*1 mapping that is insensitive to systemic bias of the underlying mapping technique.^9^ The calculation of the ECV assumes an equilibrium between the contrast concentration in the blood and the myocardium’s extracellular space after injection of an extracellular gadolinium-based contrast agent. After correcting for the haematocrit (HCT) in the blood, the ECV can be estimated from the *T*1 relaxation time changes in the myocardium and blood per the following equation:

$$\mathrm{ECV}=(1-\mathrm{HCT})\;*\;\frac{\frac{1}{\;\;\mathrm{post-contrast}\;\mathrm{myocardial}\;T1\;}-\frac{1}{\mathrm{native}\;\mathrm{myocardial}\;\;T1\;}}{\;\frac{1}{\mathrm{post-contrast}\;\mathrm{blood}\;T1}-\frac{1}{\mathrm{native}\;\mathrm{blood}\;T1}}.$$

By substituting the relaxation time *T*1 with the relaxation rate *R*1 = 1/*T*1, the formula can be simplified to:

$$\mathrm{ECV}=(1-\mathrm{HCT})\;*\;\frac{\;\mathrm{post-contrast}\;\mathrm{myocardial}\;R1-\mathrm{native}\;\mathrm{myocardial}\;\;R1}{\;\mathrm{post-contrast}\;\mathrm{blood}\;R1-\mathrm{native}\;\mathrm{blood}\;R1}.$$

As a percentage of tissue volume, ECV accurately reflects the volume range occupied by the non-cellular component of myocardial tissue and is associated with changes in the myocardial interstitium, most importantly an increase in collagen fibres. ECV reflects the intrinsic physiological properties of myocardial tissue and is relatively unaffected by various technical factors compared to the *T*1 relaxation time.^10^ A meta-analysis showed that the average of all ECV study groups at 1.5 T was 25.9% (95% CI: 25.5%, 26.3%), which was equal to the average of all study groups at 3 T of 25.9% (95% CI: 25.4%, 26.5%).^8^ Elevated ECV values have been reported in primary and systemic cardiac disease, including dilated and hypertrophic cardiomyopathy, aortic stenosis, cardiac amyloidosis, and myocardial infarction. ECV is gaining recognition as a critical biomarker of myocardial collagen content expansion;^11^ it is also referred to as ‘non-invasive’ or ‘virtual’ biopsy,^12^ and despite the lack of standardization of the acquisitions and standardized reference ranges, ECV is increasingly used in clinical practice. In sarcomere mutation carriers with hypertrophic cardiomyopathy, elevated ECV has been reported even before the development of left ventricular hypertrophy.^13^ Similarly, ECV elevations precede the development of LGE in cardiac amyloidosis, which suggests the value of ECV at earlier disease stages.^14^ ECV is also a practical prognostic factor in amyloidosis, with a hazard ratio of death of 3.85 for ECV fractions >45%.^15^ In aortic and mitral valve disease, elevations in ECV, but not *T*1 or LGE, correlate well with histologic fibrosis and, at least in aortic stenosis, track well with cardiac remodeling.^16^ Both native *T*1, ECV, and LGE have been shown to be independent predictors of cardiac death and transplantation in DCM.^17^ ECV can also be used to assess the cardiotoxicity of anthracycline chemotherapy^18,19^ and is often used in heart failure studies as a viable surrogate marker of extracellular matrix expansion.^20^ Left ventricular ECV expansion has been shown to be a strong independent predictor of AF recurrence after catheter ablation.^21^ Increased ECV was found to be associated with ventricular arrhythmia, hospitalization for heart failure, and death in tetralogy of Fallot.^22^ These findings may lead to future studies exploring the role of ECV in improving risk stratification and guiding therapeutic interventions.

### The role of HCT in ECV measurement

The HCT value is essential for ECV calculations. Generally, HCT is a low-cost blood test that can be measured with widely available laboratory equipment, either with optical or electrical methods. However, not all patients will have routine blood tests to assess their HCT before the CMR scan. Even for hospitalized patients, laboratory values of HCT may be checked routinely when admitted, but due to the high variability, the values may have already changed when the CMR scan is performed. Blood sampling at the time of the CMR scan requires point-of-care analysis equipment that is not readily available in all CMR centres, is prone to measurement errors, and disrupts the workflow.^23,24^

As mentioned before, the calculation of ECV assumes an equilibrium of the contrast agent concentration in the myocardial and blood pool extracellular space, the latter of which is calculated from the HCT. Because of the high temporal variability of HCT, it should be measured as close as possible to the CMR scan.

The Society for Cardiovascular Magnetic Resonance (SCMR) Consensus Statement recommends that the HCT should be measured within 24 h of the CMR scan because of its variability.^7^ The HCT level has been reported as highly influenced by diurnal fluctuations and may even change in a few hours.^25^ Thirup *et al*.^26^ conducted a meta-analysis exploring substantial changes in HCT in 12 studies representing 638 healthy adults, sampled at intervals ranging from 1 day to 1–2 months. They reported a relative change between two consecutive HCT measurements of ∼12%, which was unexpectedly high. Furthermore, even if HCT is obtained as close to the CMR scan as possible, HCT also changes with body posture. Engblom *et al*.^27^ reported that HCT changed by 8% when comparing blood samples drawn before (arriving at the MR department) and after CMR examination (still in supine position). This change in HCT due to change in body posture was also found by Jacob *et al*.^28^ who showed that in healthy subjects, a change of 11.0% was observed comparing a supine position to 30 min of standing. Even when measured simultaneously, the HCT can differ between peripheral venous and arterial blood.^29^

Ideally, the HCT of ventricular blood at the time of scanning would be used as a reference to calculate ECV, as the calculation of ECV is based on the *T*1 relaxation time of ventricular blood and myocardium. However, ventricular blood sampling is highly invasive and therefore not feasible for routine CMR scans.

These factors impose additional constraints and caveats on the measurement of peripheral venous blood HCT which add to the workflow, introduce errors, and ultimately limit its applicability in clinical routine work, thus imposing a barrier to the routine clinical use of ECV measurements.

## Deriving a ‘synthetic HCT’

### Relationship between the HCT and the native blood *T*1 relaxation rate (*R*1)

For the above-mentioned reasons, non-invasive ECV measurement techniques without blood sampling have gained considerable attention. Recent studies have proposed that the HCT could be estimated from the native *T*1 relaxation rate (*R*1, the inverse of the *T*1 relaxation time) of ventricular blood. The calculation is based on the linear relation of the native *R*1 of blood and the HCT^30^: the Fe II+ ions in the haemoglobin exhibit paramagnetic properties, prolonging the *R*1 of the surrounding protons. As the water in the plasma and erythrocyte cytoplasm undergoes rapid water exchange, this affects the *R*1 of the whole blood pool.^31^ A linear regression equation for the HCT can be derived from the native blood *R*1 values, allowing for a ‘synthetic’ HCT to be calculated without blood sampling. Notably, Kazuki *et al*. reported that the *T*1 relaxation time is slightly longer in arterial than venous blood.^30,31^ The reason for that is that deoxyhaemoglobin is more abundant in venous than arterial blood, the Fe III+ of which exhibits stronger paramagnetism and further reduces the *T*1 relaxation time.^31^ Hanzhang *et al*. determined blood *T*1 relaxation times at 3 T under physiological conditions, demonstrating that an increased HCT causes a reduction in blood *T*1 relaxation times; the regression coefficients for *R*1 vs. HCT were 0.52 and 0.83 for arterial and venous blood, respectively, meaning that an arterial HCT increase of 1% causes an *R*1 increase of 0.0052[s-1].^30^ These differences in arterial and venous regression parameters must be considered when choosing the measurement site for the synthetic HCT.

Compared to conventionally measured HCT, the synthetic HCT is measured during the CMR scan and is calculated from the *R*1 of the left ventricular blood, which is also the site used for ECV calculation.^32^ It therefore also avoids any potential variability stemming from differences in left ventricular to peripheral venous blood or by postural, diurnal, and day-to-day variations that might affect conventional HCT measurements.^25,26,28,29,33^

### Statistical considerations in the measurement of synthetic ECV

The parameters of the linear relationship between blood *R*1 and HCT can be estimated via linear regression. However, in doing so, several caveats have to be considered. First, to avoid ‘over-fitting’, testing of the model should not be performed on the same data that it was fitted on. This means, that the available data should be split into derivation and validation cohorts.

**Figure 1:**
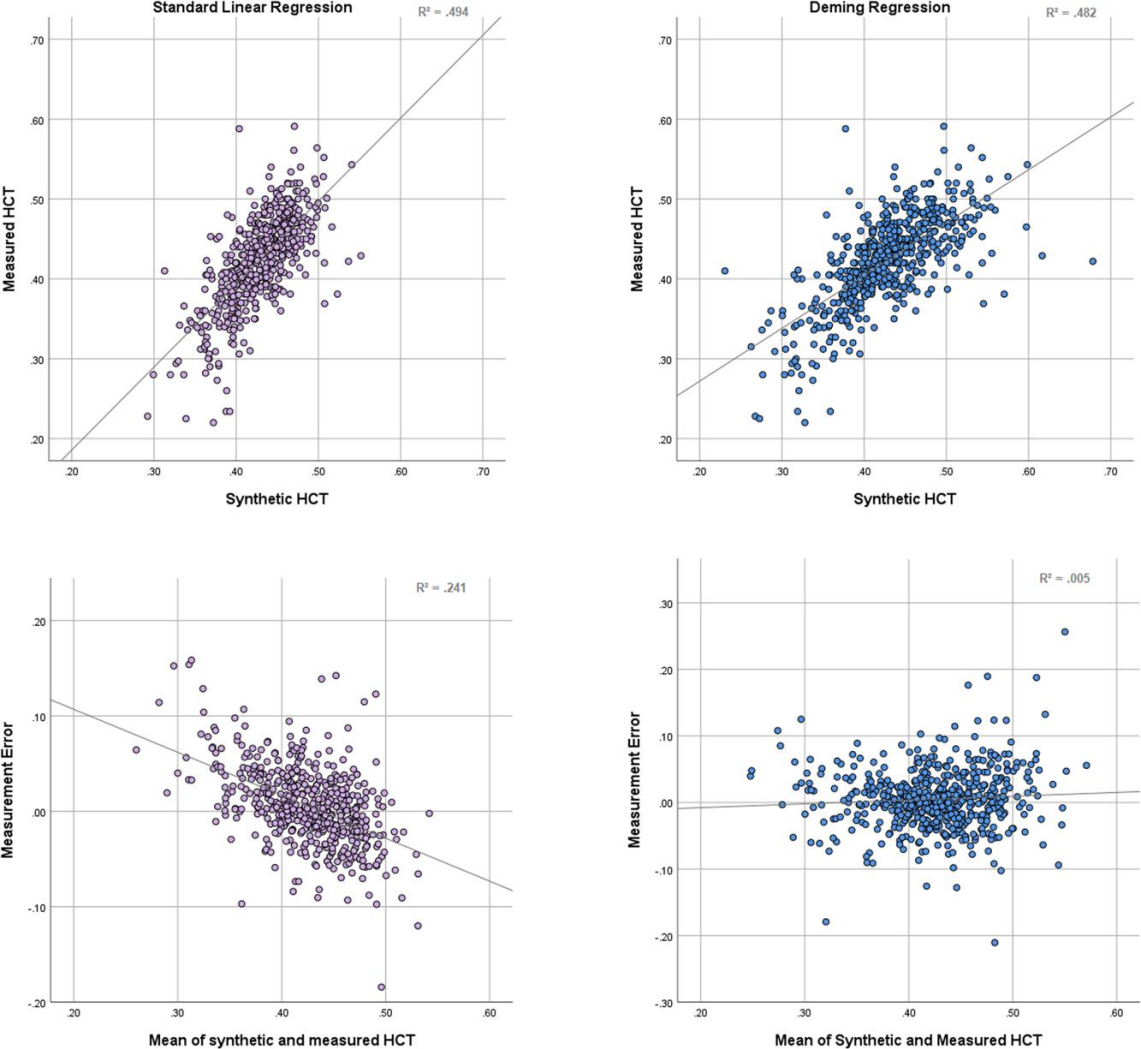
Bland–Altman analysis of measured haematocrit vs. synthetic haematocrit derived from standard linear regression (left) and Deming regression (right). The validation data set from Chen *et al*. was used. While the standard linear regression provides a slightly better fit, measured by *R*², the orthogonal regression eliminates the slope of the measurement error and is therefore more accurate at extreme values.

Second, standard linear regression relies on the assumption that the measurement error lies solely in the dependent (response) variable. Measurement error in the independent (regressor) variable will invariably cause underestimation of the regression coefficient β in standard linear regression, known as attenuation bias. This translates to an overestimation of low values and an overestimation of high values. Several sophisticated ‘errors in variables’ models have been developed to offset this effect. Of these, the Deming regression is most widely used in medical measurement comparisons and assumes measurement error in both variables, the ratio of which can be adjusted according to the data.^34^ Using a Deming regression does not necessarily improve the fit as measured by *R*² but eliminates the slope in the errors (*Figure 1*).

Third, reporting the regression coefficient *R*² of synthetic vs. standard HCT and ECV measurements is not sufficient to judge agreement of both measurements. As Bland and Altman have elaborated in their seminal 1982 paper, agreement is best assessed from the bias and limits of agreement (LoA) of both measurements, where the bias is the mean difference of both measurements and the LoA the interval into which 95% of the differences are expected to fall (usually calculated as 1.96 * SD of the difference). In any linear regression analysis, the mean of the predicted values equals the mean of the dependent values. This means that the mean synthetic HCT always equals the mean measured HCT in the derivation cohort and will be very close to the measured HCT in the validation cohort if both have been selected randomly from the same population. In this case, the bias will always be close to zero and meaningless as a measure of agreement. This leaves the LoA as the most useful parameter for the assessment of synthetic ECV.

### Sample size calculation for establishing a local model

Unfortunately, *T*1 relaxation times depend on the local setup and can vary considerably between sites and scanners. Consequently, external models provide less accuracy than models derived from a locally acquired data set. Local models can usually be derived easily from existing data with reasonable effort. The size of derivation data sets varies considerably in published models, owing in part to a lack of sample size calculations. To achieve an accurate model without waste of resources, we recommend a systematic approach as outlined by Riley *et al*.^35^ Assuming a mean HCT of 0.43 with a standard deviation of 0.05 and an *R*² of 0.428, for a model consisting of three parameters (left ventricular *R*1, sex, and field strength), a minimum derivation sample size of 237 would be necessary to provide accurate estimates of all regression parameters with little risk of over-fitting. Riley *et al*. also give recommendations regarding sample size calculations for external validation.^36^ The 95% CI for $R_{val}^{2}$ is approximately ${\hat{R}}_{val}^{2}\;\pm (1.96\times SE_{{\hat{R}}_{val}^{2}}^{2})$, where ${\hat{R}}_{val}^{2}$ is the anticipated *R*² of synthetic vs. measured ECV and $SE_{{\hat{R}}_{val}^{2}}^{2}$ its standard error. The latter is calculated as $SE_{{\hat{R}}_{val}^{2}}^{2}=\sqrt{\frac{4R_{val}^{2}(1-R_{val}^{2})}{n}}$. Assuming an *R*² of 0.88 from Chen *et al*. and an equal validation sample size of 237 leads to a CI width of 0.06, which seems acceptable. This would translate to a sample size of roughly 500 patients, equally split into derivation and validation data sets.

### Clinical research of synthetic ECV

Treibel *et al*.^37^ first proposed that synthetic HCT and ECV can be derived directly from the CMR examination itself in 2016. They plotted the relationship of conventionally measured HCT vs. the *R*1 of blood determined by two CMR pulse sequences, the MOLLI and the shortened MOLLI (ShMOLLI) recovery, at 1.5 T (Siemens MAGNETOM Avanto, Espree, and Aera, Siemens Medical Solutions, Malvern, Pennsylvania). Native blood *R*1 was linearly related to laboratory HCT with a coefficient of determination (*R*²) of 0.51 (MOLLI) and 0.45 (ShMOLLI), respectively. Despite the only moderate correlation between *R*1 and HCT, the synthetic ECV (estimated from the synthetic HCT) was closely correlated to the conventionally measured ECV (using laboratory HCT) with an *R*^2^ = 0.97 for both mapping techniques, albeit with wide LoA (±5.49% for ShMOLLI). A thorough discussion of the possible confounders can be found in the next chapter. The authors found no difference in correlation of synthetic and conventionally measured ECV and postoperative histological measurements of the collagen volume fraction in 18 aortic stenosis patients. Furthermore, synthetic and conventionally measured ECV were equally well related to the risk of hospitalization for heart failure or death in 1172 subjects in their study. The work of Treibel *et al*. provided the first comprehensive description of the accuracy and clinical suitability of synthetic ECV and is likely to enhance CMR research studies of cardiomyopathy greatly.

Fent *et al*.^38^ examined the correlation and agreement between synthetic ECV and conventional measured ECV on 1.5 T (Ingenia, Philips, Best, the Netherlands) and 3 T (Achieva dStream) Philips scanners in 2017. Their results showed that synthetic ECV values were closely correlated with conventionally measured ECV (*R*^2^ = 0.95 and *R*^2^ = 0.91 for 1.5 and 3 T, respectively). The bias between synthetic ECV and conventionally measured ECV was small with moderate LoA at 1.5 T (bias = −0.81%, LoA −4.97% to 3.35%) and 3 T (bias = −0.30%, LoA −3.92% to 3.33%). The study of Fent *et al*. further supported the practical value of synthetic ECV measurements on different scanners.

Kammerlander *et al*.^39^ examined the synthetic ECV in 513 subjects at 1.5 T (Siemens MAGNETOM Avanto). Synthetic HCT was moderately correlated with conventionally measured HCT (*R*² = 0.28), but synthetic ECV was highly correlated with measured ECV (*R*² = 0.89). Their Bland–Altman analysis showed a bias of 0.01% with LoA of −4.32% to 4.33%.

Lim *et al*.^40^ studied 143 hypertensive patients at 1.5 T (Siemens MAGNETOM Aera). They found that the model provided as an inline module (Siemens WIP#1041) by their scanner manufacturer severely underestimated the ECV especially for women (bias −8.9% for women and −4.3% for men). Their own gender-specific regression model eliminated the mean difference, as expected, but showed wide LoA of −9.41 to 9.63%. The study of Lim *et al*. highlighted both the importance of gender-specific and locally derived models in synthetic ECV measurements. However, their cohorts included only hypertensive patients, which might not be a representative population.

In 2020, Su *et al*.^33^ compared the measurement error of a locally derived synthetic HCT formula at 1.5 T (Siemens MAGNETOM Aera) with the test–retest variability of two haematocrit measurements with a median of 117 days apart. For the derived ECV measurements, they reported LoA of −3.18 to 3.60% for synthetic vs. same–day measured haematocrit and LoA of −2.75 to 2.85% for the two haematocrit measurements. Furthermore, Su *et al*. demonstrated that the error of synthetic ECV was significantly correlated with measured HCT, and relatively large ECV errors occurred when measured HCT was lowest, meaning that the difference in ECV was inversely proportional to laboratory HCT, leading to a more significant ECV error in patients with severe anaemia. This is mostly due to the attenuation bias described above. These findings suggest that synthetic ECV derived from standard linear regression should be interpreted with caution for patients with a history of anaemia, as the synthetic HCT will be overestimated and the ECV underestimated. The question remains how to reliably spot patients with anaemia without HCT testing.

Raucci *et al*.^41^ showed that the use of synthetic ECV might lead to clinical mis-categorization of paediatric and young adult patients. They assessed the accuracy of synthetic ECV in 114 children and young adults undergoing CMR at 1.5 T (Siemens MAGNETOM Avanto), using an ECV cut-off of 28.5%. Raucci *et al*. identified 23% mis-categorization using a locally derived regression equation when compared to the measured ECV. The mis-categorization was as high as 37% when applying an external published model (Treibel *et al*.), highlighting the need for locally derived models due to the known dependency of *T*1 relaxation times on the local setup.^7^ Furthermore, Shang *et al*.^42^ further illustrated that at 3 T (Siemens MAGNETOM Trio), based on their central normal cut-off value of 31.8%, despite an excellent linear regression fit, the use of synthetic ECV may lead to mis-categorization in 6–25% of patients, particularly for those with only a subtle elevation in ECV. This underlines the inadequacy of the correlation coefficient for the assessment of agreement and the need for caution when interpreting small ECV deviations. Expectedly, mis-categorization occurred mostly at values close to the cut-off. Of note, mis-categorization was not judged based on the gold standard tissue biopsy but on the conventional ECV measurement, for which there is no universal agreement on a cut-off so far.

Recently, Chen *et al*. developed and tested a model in a large data set of 1101 patients examined on 1.5 T (Philips Achieva) and 3 T (Philips Ingenia) scanners, controlling for sex and field strength.^32^ Both standard linear regression and Deming regression were performed. Standard linear regression showed narrower LoA of −4.1 to 3.7% for synthetic ECV vs. −4.6 to 4.3% for the Deming regression, but Deming regression provided less bias in anaemic patients (−1.2 ± 2.2% vs. −2.4 ± 1.7%). A validation of the standard linear regression model vs. histological analysis fraction showed similar correlation of the collagen volume with conventionally measured ECV (*R*^2^ = 0.66, *P* < 0.0001) and synthetic ECV (*R*^2^ = 0.63, *P* = 0.0001). In a cohort of amyloidosis patients, no systematic differences were found between synthetic ECV via standard linear regression and conventionally measured ECV (51.9 ± 10.9% vs. 51.1 ± 10.3% *P* = 0.18).

Overall, the aforementioned studies provide evidence of the feasibility of synthetic ECV measurements on different scanning machines, field strengths, and populations but highlight the necessity for locally derived models and prospective studies on the prognostic and diagnostic value of synthetic ECV.

### Confounders of synthetic and conventional HCT and ECV

Notably, it was found in abovementioned studies that while synthetic HCT and conventional HCT were only moderately correlated, synthetic ECV values were closely correlated with conventionally measured ECV (*Table 1*).^32,37–41^ For all studies with validation cohorts, the weighted mean of *R*² for synthetic and conventional HCT was 0.42 and for synthetic and conventional ECV 0.90.

**Table 1:** Published regression coefficients for synthetic HCT and ECV

Study	CMR scanner	*N* derivation	*N* validation	Sex	Sequence	Regression model^a^			ECV^b^
						Intercept (β0)	Slope (β1)	*R*²	*R*²
With validation cohort									
Chen^32^	Philips 1.5 T + 3 T	550	551	Mixed	MOLLI	−0.027	816.3	0.43	0.88
						+0.024 (if male)			
						−0.094 (if 1.5 T)			
Fent^38^	Philips 1.5 T	102	101	Mixed	MOLLI	−0.167	922.6	0.50	0.95
	Philips 3 T	109	109	Mixed	MOLLI	−0.071	869.7	0.46	0.92
Treibel^37^	Siemens 1.5 T	214	213	Mixed	MOLLI	−0.123	866.0	0.51	0.97
		214	213	Mixed	ShMOLLI	−0.068	727.1	0.45	0.97
Kammerlander^39^	Siemens 1.5 T	200	313	Mixed	MOLLI	−0.002	628.5	0.35	0.89
Shang^42^	Siemens 3 T	121	105	Mixed	MOLLI	0.098	562.0	0.19	0.70
Su^33^	Siemens 1.5 T	85	109	Mixed	MOLLI	0.182	971.6	0.51	0.94
Without validation cohort									
Raucci^41^	Siemens 1.5	114	—	Mixed	MOLLI	−0.213	315.1	0.16	0.82
Lim^40^	Siemens 1.5 T	143	—	Mixed	MOLLI	0.054	574.7	0.44	0.75
		53	—	Female	MOLLI	0.234	258.5	0.08	0.73
		90	—	Male	MOLLI	0.052	592.7	0.34	0.70

a Synth HCT = β_0_ + β_1_*R1_Blood_

b Correlation of synthetic vs. conventionally measured ECV

**Figure 2:**
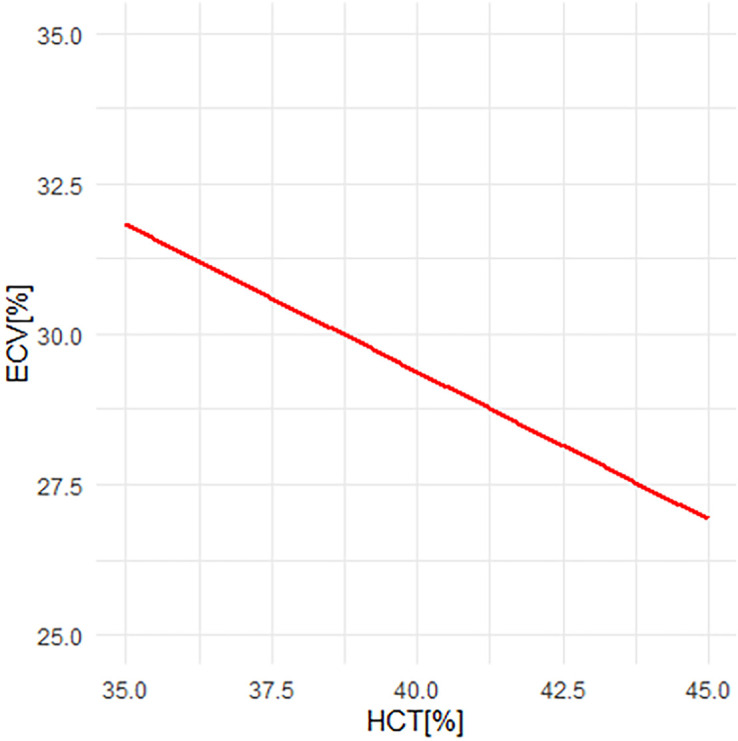
ECV vs. haematocrit (HCT) in a fictional patient. A change in HCT of 10% causes a change in ECV of ∼5%. Other parameters as follows: Post-contrast myocardial *T*1 430 ms, native myocardial *T*1 1000 ms, post-contrast blood *T*1 300 ms, native blood *T*1 1600 ms.

The close correlation between synthetic and conventional ECV, as compared to HCT, can be attributed to the four additional terms (*R*1 of myocardial and ventricular blood before and after contrast injection) that remain constant in the ECV calculation. These constants counterbalance larger changes in HCT, resulting in smaller alterations in ECV. This mitigating effect is visualized in *Figure 2*.

The only moderate correlation of synthetic and conventional HCT can be explained by several error sources. A summary of the most common influencing factors is given in *Table 2*. First, the high variability of HCT itself will influence the correlation between conventional HCT and synthetic HCT. To understand the sources of variability attributable to synthetic HCT and conventionally measured HCT, Treibel *et al*. performed two laboratory HCT measurements with an average 4-h time difference in a subset of 44 patients.^37^ They found an unexpectedly high variation of 10% between both measurements (*R*^2^ = 0.86), accounting for part of the measurement error between synthetic and measured HCT, but the generalizability of this finding is unclear due to the small sample size.

**Table 2:** Confounders of synthetic and conventional HCT

Confounders of synthetic HCT	Confounders of conventional HCT
Anaemia (standard linear regression)	Diurnal variation
Sex	Day-to-day variation
Young age	Postural variation
Hypoxia	Arteriovenous variation
Iron overload	
Abnormal mean corpuscular haemoglobin (severe iron, folate, or B12 deficiency)	
Scanner (when using external model)	

Second, factors affecting *T*1 relaxation times in the blood may also affect this relationship. Common influencing factors of *T*1 relaxation times include physiological changes such as total serum protein, temperature, and haemoglobin oxygenation, as well as technical issues including magnetic field heterogeneity, pulse sequence parameters, efficiency of inversion pulses, magnetization transfer effects, and fitting algorithms.^43,44^ Third, the estimation of the synthetic HCT is based on the relaxation properties of iron. While most blood iron is bound to haemoglobin and haemoglobin and HCT are closely correlated, the mean corpuscular haemoglobin concentration (MCHC) can vary and introduce noise, especially in patients with deficiencies in iron, folate or vitamin B12.^45^ Additionally, blood iron outside of haemoglobin has been reported to have a substantial effect on *T*1 relaxation times,^46^ and in patients with iron overload, particularly in thalassaemic patients, the *R*1/HCT relationship may be broken.^46^ The contribution of other biological variables such as lipids need further study. In addition, blood flow artefacts in the ventricular cavity may lead to unexpected variations. These factors may contribute to measurement variability and thereby reduce correlation between conventionally measured and synthetic HCT.

Fourth, HCT is conventionally measured in the peripheral venous blood, whereas the synthetic HCT regression equation is derived from the *T*1 relaxation time of left ventricular arterial blood. The HCT difference between peripheral and central blood as well as arterial and venous blood may also introduce noise, given that the *T*1 relaxation time of blood is usually measured in the left ventricle and HCT in peripheral venous blood.

Fifth, *T*1 relaxation times are dependent on the field strength, vendor, and acquisition technique. Using a formula for synthetic HCT derived under a different setup may lead to systematic errors in synthetic ECV, as encountered by Raucci *et al*.^41^ This might seem counterintuitive, because conventional ECV measurements are quite reproducible between setups. The reason for the latter is that the ECV formula uses the differences in *T*1 relaxation rates (the inverse of the *T*1 relaxation times) before and after contrast agent, so that systematic differences even out. The synthetic HCT is calculated using absolute *T*1 relaxation rates, not differences, so that systematic differences will produce systematic errors in the synthetic HCT and therefore the synthetic ECV.

### Choosing the optimal cut-off value for synthetic ECV

**Figure 3:**
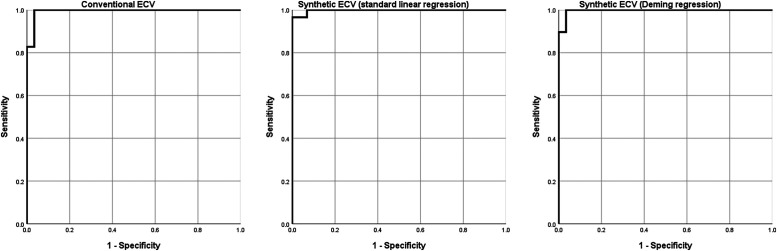
ROC curves for the diagnosis of cardiac amyloidosis using conventional ECV, synthetic ECV using standard linear regression, and synthetic ECV using a Deming regression. Data set from Chen *et al*.^32^

It should be noted that there is no consensus on the optimal cut-off value for conventionally measured ECV.^7^ Published upper limits of normal range from 29 to 36%.^47^ As synthetic ECV does not show systemic bias when using locally derived formulas, similar cut-off values can be used as for conventionally measured ECV. Chen *et al*. found a misclassification of 6% when using a cut-off of 29.5% for synthetic and 30% for conventionally measured ECV. Furthermore, they found no significant difference in values for measured and synthetic haematocrit in a cohort of 29 amyloidosis patients (mean ECV 51.1 ± 10.3% vs. 51.9 ± 10.9%). A receiver operator characteristic (ROC) analysis for the diagnosis of amyloidosis based on the data by Chen *et al*. is given in *Figure 3*.

### Clinical applications and prospects of synthetic ECV

Previous investigators have demonstrated on different platforms that synthetic ECV obtained by synthetic HCT is feasible, and synthetic ECV values correlate closely with conventionally measured ECV. While the LoA were rather wide, this might also be attributable to variations in conventional HCT measurements. Limited data on outcome and histological validation suggest diagnostic equivalence between conventional and synthetic HCT. A great advantage of synthetic ECV lies in its ability to offer the advantages of ECV measurements to CMR laboratories without access to timely HCT sampling.

However, several caveats need to be further evaluated before widespread clinical application. First, while synthetic ECV methods can be applied to multiple centres and multiple scanning platforms, each centre will need to derive its formula for the *R*1/HCT relationship because *T*1 relaxation times are known to be setup dependent. We recommend using a Deming regression to avoid attenuation bias, but consultation with your local statistician is advisable. Second, age- and gender-specific formulas should be considered due to physiological differences in blood composition. Third, concerns regarding mis-categorization still need to be addressed in future studies. Particular attention needs to be given to patients with anaemia and children and adolescents. In large retrospective study cohorts where HCT was not measured, synthetic ECV remains of high utility. It is still recommended that standard HCT values be obtained whenever feasible. Finally, most studies so far have focused on the accuracy of synthetic ECV compared to conventional ECV, rather than the gold standard of tissue biopsy. While histological validation in small subgroups by Treibel *et al*. and Chen *et al*. showed similar correlations between synthetic and conventional ECV and tissue biopsy results, large-scale validations of synthetic ECV based on tissue biopsy and clinical outcome are lacking.

## Conclusion

Synthetic HCT and ECV can provide a non-invasive quantitative measurement of the myocardium’s extracellular space when timely HCT measurements are not available and large alterations in ECV are expected, such as in cardiac amyloidosis. Due to the dependency of *T*1 relaxation times on the local setup, calculation of local formulas is recommended, which can be easily performed using available data. Open questions regarding its accuracy remain, and further studies on its prognostic and diagnostic value are necessary.

## Data Availability

The data underlying *Figures 1* and *3* are available upon reasonable request.

## Lead author biography

Patrick Doeblin is the deputy head of cardiovascular MRI at the Deutsches Herzzentrum der Charité with board certifications in cardiology, CMR, and medical informatics. He graduated from the Charité University Medicine Berlin and completed his general cardiology training at the Charité Campus Benjamin Franklin and the German Heart Centre Berlin. His research interests are CMR tissue characterization and computational approaches to imaging.

## References

[1] Gibb AA, Lazaropoulos MP, Elrod JW. Myofibroblasts and fibrosis: mitochondrial and metabolic control of cellular differentiation. Circ Res 2020;127:427–47. 32673537 10.1161/CIRCRESAHA.120.316958 PMC7982967

[2] Aretz HT, Billingham ME, Edwards WD, Factor SM, Fallon JT, Fenoglio JJ Jr et al Myocarditis. A histopathologic definition and classification. Am J Cardiovasc Pathol 1987;1:3–14. 3455232

[3] Beltrami CA, Finato N, Rocco M, Feruglio G, Puricelli C, Cigola E et al The cellular basis of dilated cardiomyopathy in humans. J Mol Cell Cardiol 1995;27:291–305. 7760353 10.1016/s0022-2828(08)80028-4

[4] Pucci A, Aimo A, Musetti V, Barison A, Vergaro G, Genovesi D et al Amyloid deposits and fibrosis on left ventricular endomyocardial biopsy correlate with extracellular volume in cardiac amyloidosis. J Am Heart Assoc 2021;10:e020358. 34622675 10.1161/JAHA.120.020358 PMC8751897

[5] Verdonschot JAJ, Hazebroek MR, Derks KWJ, Barandiarán Aizpurua A, Merken JJ, Wang P et al Titin cardiomyopathy leads to altered mitochondrial energetics, increased fibrosis and long-term life-threatening arrhythmias. Eur Heart J 2018;39:864–73. 29377983 10.1093/eurheartj/ehx808

[6] Weber KT, Brilla CG. Pathological hypertrophy and cardiac interstitium. Fibrosis and renin-angiotensin-aldosterone system. Circulation 1991;83:1849–65. 1828192 10.1161/01.cir.83.6.1849

[7] Messroghli DR, Moon JC, Ferreira VM, Grosse-Wortmann L, He T, Kellman P et al Clinical recommendations for cardiovascular magnetic resonance mapping of *T*1, *T*2, *T*2* and extracellular volume: a consensus statement by the Society for Cardiovascular Magnetic Resonance (SCMR) endorsed by the European Association for Cardiovascular Imaging (EACVI). J Cardiovasc Magn Reson 2017;19:75. 28992817 10.1186/s12968-017-0389-8 PMC5633041

[8] Gottbrecht M, Kramer CM, Salerno M. Native *T*1 and extracellular volume measurements by cardiac MRI in healthy adults: a meta-analysis. Radiology 2019;290:317–26. 30422092 10.1148/radiol.2018180226 PMC6358026

[9] Robinson AA, Chow K, Salerno M. Myocardial *T*1 and ECV measurement: underlying concepts and technical considerations. JACC Cardiovasc Imaging 2019;12:2332–44. 31542529 10.1016/j.jcmg.2019.06.031 PMC7008718

[10] Moon JC, Messroghli DR, Kellman P, Piechnik SK, Robson MD, Ugander M et al Myocardial *T*1 mapping and extracellular volume quantification: a Society for Cardiovascular Magnetic Resonance (SCMR) and CMR working group of the European Society of Cardiology consensus statement. J Cardiovasc Magn Reson 2013;15:92. 24124732 10.1186/1532-429X-15-92 PMC3854458

[11] aus dem Siepen F, Buss SJ, Messroghli D, Andre F, Lossnitzer D, Seitz S et al *T*1 mapping in dilated cardiomyopathy with cardiac magnetic resonance: quantification of diffuse myocardial fibrosis and comparison with endomyocardial biopsy. Eur Heart J Cardiovasc Imaging 2015;16:210–6. 25246502 10.1093/ehjci/jeu183

[12] Kramer CM, Chandrashekhar Y, Narula J. *T*1 mapping by CMR in cardiomyopathy: a noninvasive myocardial biopsy? JACC Cardiovasc Imaging 2013;6:532–4. 23579019 10.1016/j.jcmg.2013.02.002

[13] Ho CY, Abbasi SA, Neilan TG, Shah Ravi V., Chen Y, Heydari B et al *T*1 measurements identify extracellular volume expansion in hypertrophic cardiomyopathy sarcomere mutation carriers with and without left ventricular hypertrophy. Circ Cardiovasc Imaging 2013;6:415–22. 23549607 10.1161/CIRCIMAGING.112.000333 PMC3769196

[14] Fontana M, Pica S, Reant P, Abdel-Gadir A, Treibel TA, Banypersad SM et al Prognostic value of late gadolinium enhancement cardiovascular magnetic resonance in cardiac amyloidosis. Circulation 2015;132:1570–9. 26362631 10.1161/CIRCULATIONAHA.115.016567 PMC4606985

[15] Banypersad SM, Fontana M, Maestrini V, Sado DM, Captur G, Petrie A et al *T*1 mapping and survival in systemic light-chain amyloidosis. Eur Heart J 2015;36:244–51. 25411195 10.1093/eurheartj/ehu444 PMC4301598

[16] de Meester de Ravenstein C, Bouzin C, Lazam S, Boulif J, Amzulescu M, Melchior J et al Histological validation of measurement of diffuse interstitial myocardial fibrosis by myocardial extravascular volume fraction from Modified Look–Locker imaging (MOLLI) *T*1 mapping at 3 T. J Cardiovasc Magn Reson 2015;17:48. 10.1186/s12968-015-0150-0 PMC4464705 26062931

[17] Li S, Zhou D, Sirajuddin A, He J, Xu J, Zhuang B et al *T*1 mapping and extracellular volume fraction in dilated cardiomyopathy: a prognosis study. JACC Cardiovasc Imaging 2022;15:578–90. 34538631 10.1016/j.jcmg.2021.07.023

[18] Neilan TG, Coelho-Filho OR, Shah RV, Feng JH, Pena-Herrera D, Mandry D et al Myocardial extracellular volume by cardiac magnetic resonance imaging in patients treated with anthracycline-based chemotherapy. Am J Cardiol 2013;111:717–22. 23228924 10.1016/j.amjcard.2012.11.022 PMC3578020

[19] Melendez GC, Jordan JH, D'Agostino RB Jr, Vasu S, Hamilton CA, Hundley WG. Progressive 3-month increase in LV myocardial ECV after anthracycline-based chemotherapy. JACC Cardiovasc Imaging 2017;10:708–9. 27544895 10.1016/j.jcmg.2016.06.006 PMC7890530

[20] Schelbert EB, Piehler KM, Zareba KM, Moon JC, Ugander M, Messroghli DR et al Myocardial fibrosis quantified by extracellular volume is associated with subsequent hospitalization for heart failure, death, or both across the Spectrum of ejection fraction and heart failure stage. J Am Heart Assoc 2015;4:e002613. 26683218 10.1161/JAHA.115.002613 PMC4845263

[21] Li S, Zhao L, Ma X, Bai R, Tian J, Selvanayagam JB. Left ventricular fibrosis by extracellular volume fraction and the risk of atrial fibrillation recurrence after catheter ablation. Cardiovasc Diagn Ther 2019;9:578–85. 32038947 10.21037/cdt.2019.12.03 PMC6987514

[22] Chen CA, Dusenbery SM, Valente AM, Powell AJ, Geva T. Myocardial ECV fraction assessed by CMR is associated with type of hemodynamic load and arrhythmia in repaired tetralogy of fallot. JACC Cardiovasc Imaging 2016;9:1–10. 26684969 10.1016/j.jcmg.2015.09.011

[23] Maslow A, Bert A, Singh A, Sweeney J. Point-of-care hemoglobin/hematocrit testing: comparison of methodology and technology. J Cardiothorac Vasc Anesth 2016;30:352–62. 27013121 10.1053/j.jvca.2015.11.010

[24] Kim WH, Lee HC, Ryu HG, Chung E-J, Kim B, Jung H et al Reliability of point-of-care hematocrit measurement during liver transplantation. Anesth Analg 2017;125:2038–44. 28537971 10.1213/ANE.0000000000002109

[25] Sennels HP, Jorgensen HL, Hansen AL, Goetze JP, Fahrenkrug J. Diurnal variation of hematology parameters in healthy young males: the Bispebjerg study of diurnal variations. Scand J Clin Lab Invest 2011;71:532–41. 21988588 10.3109/00365513.2011.602422

[26] Thirup P. Haematocrit: within-subject and seasonal variation. Sports Med 2003;33:231–43. 12656642 10.2165/00007256-200333030-00005

[27] Engblom H, Kanski M, Kopic S, Nordlund D, Xanthis CG, Jablonowski R et al Importance of standardizing timing of hematocrit measurement when using cardiovascular magnetic resonance to calculate myocardial extracellular volume (ECV) based on pre- and post-contrast *T*1 mapping. J Cardiovasc Magn Reson 2018;20:46. 29950178 10.1186/s12968-018-0464-9 PMC6022290

[28] Jacob G, Raj SR, Ketch T, Pavlin B, Biaggioni I, Ertl AC et al Postural pseudoanemia: posture-dependent change in hematocrit. Mayo Clin Proc 2005;80:611–4. 15887428 10.4065/80.5.611

[29] Mokken FC, van der Waart FJM, Henny CP, Goedhart PT, Gelb AW. Differences in peripheral arterial and venous hemorheologic parameters. Ann Hematol 1996;73:135–7. 8841101 10.1007/s002770050214

[30] Lu H, Clingman C, Golay X, van Zijl PC. Determining the longitudinal relaxation time (*T*1) of blood at 3.0 Tesla. Magn Reson Med 2004;52:679–82. 15334591 10.1002/mrm.20178

[31] Shimada K, Nagasaka T, Shidahara M, Machida Y, Tamura H. In vivo measurement of longitudinal relaxation time of human blood by inversion-recovery fast gradient-echo MR imaging at 3 T. Magn Reson Med Sci 2012;11:265–71. 10.2463/mrms.11.265 23269013

[32] Chen W, Doeblin P, Al-Tabatabaee S, Klingel K, Tanacli R, Jakob Weiß K et al Synthetic extracellular volume in cardiac magnetic resonance without blood sampling: a reliable tool to replace conventional extracellular volume. Circ Cardiovasc Imaging 2022;15:e013745. 35360924 10.1161/CIRCIMAGING.121.013745 PMC9015035

[33] Su MY, Huang YS, Niisato E, Chow K, Juang J-MJ, Wu C-K et al Is a timely assessment of the hematocrit necessary for cardiovascular magnetic resonance-derived extracellular volume measurements? J Cardiovasc Magn Reson 2020;22:77. 33250055 10.1186/s12968-020-00689-x PMC7702722

[34] Deming WE. Statistical Adjustment of Data. New York: Whiley; 1943.

[35] Riley RD, Ensor J, Snell KIE, Harrell FE, Martin GP, Reitsma JB et al Calculating the sample size required for developing a clinical prediction model. BMJ 2020;368:m441. 32188600 10.1136/bmj.m441

[36] Archer L, Snell KIE, Ensor J, Hudda MT, Collins GS, Riley RD. Minimum sample size for external validation of a clinical prediction model with a continuous outcome. Stat Med 2021;40:133–46. 33150684 10.1002/sim.8766

[37] Treibel TA, Fontana M, Maestrini V, Castelletti S, Rosmini S, Simpson J et al Automatic measurement of the myocardial interstitium: synthetic extracellular volume quantification without hematocrit sampling. JACC Cardiovasc Imaging 2016;9:54–63. 26762875 10.1016/j.jcmg.2015.11.008

[38] Fent GJ, Garg P, Foley JRJ, Swoboda PP, Dobson LE, Erhayiem B et al Synthetic myocardial extracellular volume fraction. JACC Cardiovasc Imaging 2017;10:1402–4. 28216005 10.1016/j.jcmg.2016.12.007

[39] Kammerlander AA, Duca F, Binder C, Aschauer S, Zotter-Tufaro C, Koschutnik M et al Extracellular volume quantification by cardiac magnetic resonance imaging without hematocrit sampling: ready for prime time? Wien Klin Wochenschr 2018;130:190–6. 28980127 10.1007/s00508-017-1267-y PMC5978936

[40] Lim EH, Le TT, Bryant J, Chung Y-C, Su B, Gan J et al Importance of sex-specific regression models to estimate synthetic hematocrit and extracellular volume fraction. JACC Cardiovasc Imaging 2018;11:1366–7. 29454764 10.1016/j.jcmg.2017.11.035

[41] Raucci FJ J, Parra DA, Christensen JT, Hernandez LE, Markham LW, Xu M et al Synthetic hematocrit derived from the longitudinal relaxation of blood can lead to clinically significant errors in measurement of extracellular volume fraction in pediatric and young adult patients. J Cardiovasc Magn Reson 2017;19:58. 28768519 10.1186/s12968-017-0377-z PMC5541652

[42] Shang Y, Zhang X, Zhou X, Wang J. Extracellular volume fraction measurements derived from the longitudinal relaxation of blood-based synthetic hematocrit may lead to clinical errors in 3 T cardiovascular magnetic resonance. J Cardiovasc Magn Reson 2018;20:56. 30089499 10.1186/s12968-018-0475-6 PMC6083590

[43] Kellman P, Arai AE, Xue H. *T*1 and extracellular volume mapping in the heart: estimation of error maps and the influence of noise on precision. J Cardiovasc Magn Reson 2013;15:56. 23800276 10.1186/1532-429X-15-56 PMC3702513

[44] Chow K, Flewitt JA, Green JD, Pagano JJ, Friedrich MG, Thompson RB. Saturation recovery single-shot acquisition (SASHA) for myocardial T(1) mapping. Magn Reson Med 2014;71:2082–95. 23881866 10.1002/mrm.24878

[45] Adeli K, Raizman JE, Chen Y, Higgins V, Nieuwesteeg M, Abdelhaleem M et al Complex biological profile of hematologic markers across pediatric, adult, and geriatric ages: establishment of robust pediatric and adult reference intervals on the basis of the Canadian Health Measures Survey. Clin Chem 2015;61:1075–86. 26044509 10.1373/clinchem.2015.240531

[46] Rosmini S, Bulluck H, Abdel-Gadir A, Treibel TA, Culotta V, Thompson R et al The effect of blood composition on *T*1 mapping. JACC Cardiovasc Imaging 2019;12:1888–90. 31103584 10.1016/j.jcmg.2019.03.018

[47] Kawel-Boehm N, Hetzel SJ, Ambale-Venkatesh B, Captur G, Francois CJ, Jerosch-Herold M et al Reference ranges (“normal values”) for cardiovascular magnetic resonance (CMR) in adults and children: 2020 update. J Cardiovasc Magn Reson 2020;22:87. 33308262 10.1186/s12968-020-00683-3 PMC7734766

